# Social Undermining and Promotive Voice: The Moderating Effects of Procedural Justice

**DOI:** 10.3390/bs14060447

**Published:** 2024-05-25

**Authors:** Abdulkhamid Komil ugli Fayzullaev, Soo Young Shin

**Affiliations:** School of Business, Yeungnam University, Gyeongsan 38541, Republic of Korea; fayzziyo@yu.ac.kr

**Keywords:** social undermining, supervisor social undermining, procedural justice, promotive voice

## Abstract

Organizations are increasingly depending on their employees to contribute suggestions aimed at enhancing organizational processes, boosting overall efficiency, and fostering innovation. However, some factors might hinder employees from expressing their thoughts. While there is evidence suggesting an effect of supervisor social undermining behavior on employee voice, the impact on innovative types of voice, specifically promotive voice, remains uncertain. This study aims to explore the association between supervisor social undermining and employee promotive voice. Moreover, this research investigates how employee perceptions of procedural justice moderate this relationship, utilizing the Conservation of Resources theory. Data were collected from 115 highly skilled employees, and hierarchical regression analysis was employed to assess the proposed hypotheses. The findings suggest that when individuals encounter social undermining behavior from their supervisor, they are less inclined to engage in promotive voice behavior. Interestingly, the results indicate that this relationship becomes stronger when individuals possess higher perceptions of procedural justice. To the best of our knowledge, this study is the first to investigate the impact of supervisor social undermining on promotive voice while considering procedural justice as a moderator in this relationship. The findings of this study suggest several theoretical and practical implications and provide directions for future research.

## 1. Introduction

Organizations are increasingly relying on employees’ contributions of their suggestions to enhance processes, efficiency, and innovation within the organization [1,2]. Employees are considered a precious source of creativity and innovation within organizations [3]. The way employees can contribute to the innovation and successful adjustment of their organizations to the dynamic business environment is by utilizing their “voice”—which involves sharing constructive viewpoints, concerns, or ideas regarding work-related matters [4]. One innovative form of employee expression of ideas and suggestions is promotive voice, which intends to improve and transform the existing state of affairs [5]. Promotive voice entails employees taking a proactive approach to suggesting beneficial initiatives and voicing their concerns aimed at improving procedures within the work unit [6]. Recognizing the vital importance of employees’ promotive voice in boosting organizational efficiency and innovation, we believe that it is crucial to investigate the barriers that hinder employees’ willingness to communicate their suggestions and ideas on workplace matters.

Several prior studies have pointed out that supervisors’ behavior and characteristics significantly influence subordinates’ decisions to voice their opinions or stay quiet [7,8,9]. Specifically, studies examining employee voice have investigated the impact of various supervisor mistreatment behaviors, such as abusive supervision [10,11], supervisor incivility [9,12], ostracism [13,14], and social undermining [15,16]. However, among various forms of mistreatment, supervisor social undermining has not been studied extensively regarding its effects on employee voice. Social undermining entails intentional behavior aimed at hindering the development and maintenance of favorable interpersonal relationships, success in professional endeavors, and a positive reputation [17]. Prior studies [15,16] have explored supervisor-driven social undermining and how it influences employees’ willingness to express work-related concerns. For example, the research conducted by Jung and Yoon [16] examined how supervisor social undermining affects various forms of employee voice, including acquiescent, defensive, and prosocial voices. Although several studies have highlighted the significance of the promotive employee voice in facilitating innovative outcomes within an organization [18,19], to the best of our knowledge, the influence of supervisor social undermining on employees’ decisions to express their promotive voices has not yet been investigated. Hence, this study examined the influence of supervisor social undermining on employees’ promotive voices.

Moreover, promotive voices endeavor to change and enhance the status quo. Consequently, employees may perceive this form of expressing their views as risky, especially in cases where their superiors exhibit behaviors that undermine social interactions. When individuals become aware that sharing their opinions may involve certain uncertainties or impact their interpersonal relationships, they may experience reluctance to voice their thoughts [20]. Hsiung’s [21] study highlighted that social context and climate serve as crucial indicators for individuals to assess the benefits and drawbacks of voice behavior. In other words, social context and climate play a role in either enabling or restraining individuals’ willingness to voice their opinions. According to Tangirala and Ramanujam [22], a climate of procedural justice influences whether employees choose to voice their opinions or stay silent. Within a workplace characterized by strong procedural justice, individuals are subject to fair treatment [23]. When individuals perceive a strong sense of procedural justice, they are confident that their recommendations will be acknowledged and not lead to unethical treatment. However, in work environments characterized by weak procedural justice, employees perceive high risks [21]. Based on the Conservation of Resources (COR) theory [24], scholars [25] indicated that individuals experiencing stressful situations tend to strategically utilize their voice to protect or obtain resources. We propose that when individuals encounter stressful situations, such as social undermining from their supervisors, they evaluate procedural justice within the organization to determine the extent to which they choose to voice their concerns. A strong perception of procedural justice diminishes perceived risks and encourages an increased voice. Conversely, a weak perception of procedural justice acts as a barrier, preventing employees from expressing their suggestions. Hence, the subsequent goal of our study was to investigate how employee procedural justice perception moderates the link between supervisors’ social undermining and employees’ promotive voices.

## 2. Literature Review and Hypothesis Development

### 2.1. Social Undermining

Social undermining is defined as “a behavior intended to hinder, over time, the ability to establish and maintain positive interpersonal relationships, work-related success, and favorable reputation” [17] (p. 332). Hershcovis [26] explained the distinction between social undermining and other forms of mistreatment in the workplace. First, the construct considers the intent of the perpetrator, unlike incivility, where the intent is uncertain. Second, the construct highlights the disruption of relationships in the workplace, meaning that third-party attitudes and behaviors toward victims are affected. Finally, the definition of social undermining makes specific assumptions about the consequences, such as hindering the victim’s reputation, negatively impacting their ability to succeed in their job, and disrupting their social relationships [26].

Supervisor social undermining is employees’ perception that their supervisors engage in behaviors that impede their performance, hinder the development of favorable workplace relationships, and undermine their reputation [27]. In work environments, supervisors are frequently regarded as the key sources of undermining [17]. Supervisors can demonstrate social undermining behavior in both overt and subtle ways, such as belittling subordinates or withholding crucial information. Other possible forms could involve making followers feel inadequate, disregarding their presence, engaging in gossip about them, and spreading rumors behind their backs.

The effects of social undermining on reputation and relationships may vary depending on its form [17]. Some forms of undermining, such as making derogatory remarks, rejecting someone outright, or ridiculing, are direct and can harm relationships and reputations. Indirect forms of undermining, like withholding information or neglecting to advocate for someone, can also be effective, but perpetrators may try to conceal the true nature of their behavior by presenting it as accidental [28]. Social undermining can take both verbal and physical forms. Verbal undermining includes making derogatory comments, providing silent treatment, and not sharing important information. Physical undermining can involve not providing the promised resources or intentionally impeding work progress to detrimentally affect the target. Interestingly, if a behavior is not seen as an intentional attempt to harm a target, it is not considered undermining [17]. Therefore, if someone believes that a supervisor is not doing his/her job or providing the necessary information because of personal stress or illness, they will not view these actions as undermining. Similarly, a rude comment made by a supervisor who says they are going through a tough time, such as a divorce, may be seen as inappropriate or stress-related but not undermining.

Supervisor social undermining may occur when employees demonstrate low efficiency, make mistakes, experience discord with their supervisor’s character, or engage in interpersonal conflict [29]. Individuals are sensitive to instances of social undermining originating from supervisors in their work environments. This is because relationships with one’s supervisor play a vital role in the workplace. After all, they significantly impact both performance and reputation [17]. Previous research has suggested that the social undermining behavior displayed by supervisors can negatively affect working conditions. For instance, increased perceptions of being subjected to undermining behavior can generate substantial adverse feelings. This is particularly true when such behavior becomes excessively widespread, resulting in an extremely undesirable work environment for those targeted [16]. Social undermining leads to significant disruptions in employees’ social relationships because they perceive deliberate targeting by their supervisors [30], thereby making the completion of their tasks particularly challenging [31]. Given that such behaviors can trigger feelings of confusion and perceived threat among employees, understanding employees’ reactions to supervisors’ social undermining is crucial [16].

### 2.2. Employee Voice

Voice pertains to the expression of constructive viewpoints, recommendations, or considerations regarding matters related to work [32]. Employee voice involves discretionary actions in which employees participate in sharing constructive ideas and opinions [33]. This type of voice is discretionary, not specified in the job description, and can take various forms. Some scholars have studied employee voice as a unitary construct [34], whereas other researchers have considered voice as a multidimensional construct and have proposed various forms, including acquiescent, defensive, and prosocial voices [4]. The acquiescent voice entails expressing ideas and opinions out of resignation, the defensive voice involves sharing ideas to protect one from negative consequences, and the prosocial voice involves offering constructive solutions with cooperative intentions [4]. Subsequently, considering voice as a multidimensional concept, Liang and colleagues [6] introduced the notions of promotive and prohibitive voices, which have gained significant traction in scholarly investigations [9,11,35,36]. Promotive voice refers to employees expressing novel ideas or suggestions to enhance the overall operation of their work unit or organization, whereas prohibitive voice involves employees expressing concerns regarding work practices, incidents, or employee behavior that could be detrimental to their organization [6]. This study focuses on promotive voices. A promotive type of voice is considered innovative, as it aims to enhance the existing state of affairs [5]. Because this form of voice challenges the existing norms, employees might perceive it as risky behavior, particularly when their superiors display behaviors that undermine social interaction.

Liang and colleagues [6] state that promotive voice involves individuals taking a proactive approach to proposing beneficial projects and expressing concerns to enhance the procedures within their work unit. A promotive voice is seen as having good intentions, as employees aim to improve the organization [5]. Promotive voice is a pro-social behavior assisting teams’ adjustment and achievement of successful outcomes in a changing environment [19]. Promotive suggestions are typically positive and are presented as potential improvements [6]. Essentially, the core idea behind promotive voice is that it conveys a future-oriented perspective and focuses on long-term enhancement and innovation [6,18]. Moreover, the promotive voice instigates changes in the present work environment with a forward-looking perspective [6] and aims to help teams adjust to changing conditions and achieve successful and innovative outcomes [19]. Given that organizations today require innovative ideas from employees to drive innovation [37], it is essential to comprehend the factors associated with employees’ promotive voice to boost organizational innovation and performance.

### 2.3. Supervisor Social Undermining and Employee Promotive Voice

Recent studies investigating voice behavior have provided empirical evidence that supervisors play a significant role in directly influencing the extent to which subordinates are willing to engage in voice behavior [8,38,39]. Supervisor behavior’s impact on employee behavior can be explained through a social exchange framework. Social exchange theory [40] suggests that individuals are involved in a social process of reciprocating actions with one another. In social exchanges, when encountering favorable initial behaviors, recipients are inclined to respond in a similar way by displaying more positive responses in return [41]. Studies centered on social exchange relationships within the workplace indicate that employees exhibit extra-role behaviors as a means of responding to positive leader-member exchange relationships [42]. Subordinates evaluate the behavior of their supervisors and the way they have been treated by them before voicing their opinions on workplace issues. When subordinates observe positive treatment and gains from their superiors, they often build trust in their supervisors, which in turn prompts them to reciprocate by offering suggestions to enhance their work environment [9]. Several studies have indicated that employees have a higher tendency to respond to positive treatment by others by participating in acts of organizational citizenship, such as voicing their opinions [4,43].

Conversely, unfavorable social interactions within the workplace can impede willingness to share knowledge. Drawing from the principles of social exchange theory [40], social undermining is identified as a detrimental form of interpersonal behavior, resulting in negative social dynamics within the workplace [17]. Social undermining hinders the target’s capacity to develop and uphold positive social interactions within the workplace [26]. Since interpersonal relationships hold significant importance in work settings, supervisors engaging in social undermining can impede followers’ willingness to share their knowledge. This occurs because behaviors that undermine colleagues in the workplace are perceived as violating the established norms of professional interactions, showcasing a disregard for and absence of consideration for others [44]. Also, engaging in undermining behaviors induces distress, prompting targets to harbor doubts about their social relationships with those responsible [45].

In line with the social exchange theory [40], if a subordinate encounters hostile treatment from their supervisor, it’s likely they will react with unfavorable behavior or responses, driven by the norm of negative reciprocity [46]. Although individuals tend to react similarly to the way they are treated [41], they may be unable to reciprocate in the same manner when confronted with interpersonal mistreatment like social undermining from their supervisor. That is because in relationships where there’s a power imbalance, like that between a supervisor and a subordinate, the less influential individual is constrained in their capacity to respond to unfavorable treatment [47]. As a result, subordinates are inclined to respond to negative treatment from their supervisor by reducing their positive organizational citizenship behaviors, such as promotive voice. In other words, if employees encounter socially undermining behavior from their supervisors, they avoid proposing constructive, work-related ideas or opinions. Therefore, we propose the following hypothesis:

**Hypothesis** **1.** *Supervisor social undermining is negatively related to employee promotive voice.*

### 2.4. Moderating Role of Procedural Justice

Procedural justice refers to how employees perceive organizational authority in terms of making decisions that not only offer control over the process but also maintain consistency, accuracy, correctability, and the elimination of any prejudice [48]. Previous studies have demonstrated that procedural justice significantly contributes to fostering positive work attitudes and organizational citizenship behaviors [49,50]. Indeed, a workplace characterized by fairness can actively enhance individuals’ motivation to express their suggestions in the workplace [51]. Individuals evaluate the cost and benefits associated with participating in voice behavior, and the social environment and climate play vital roles in facilitating or limiting individuals’ propensity to share their opinions [21]. Specifically, perceptions of procedural justice aid individuals in organizations by fostering a sense that their contributions are sought and valued by authorities during the decision-making process [52]. Hence, we assume that the way employees perceive procedural justice has a crucial role in the link between supervisor social undermining and employees’ promotive voices.

While the impact of supervisor mistreatment on employee voice behavior aligns with the social exchange framework [53,54], the role of procedural justice in moderating this relationship can be explained through the COR theory [24]. The COR theory [24] is a psychological framework that explains how individuals strive to acquire, protect, and build resources to cope with stress and achieve well-being. Building upon the COR theory [24], social undermining is considered a stressful situation that hinders an individual’s access to internal organizational resources, including positive workplace interpersonal relationships [55].

Based on COR theory [24], Ng and Feldman [25] suggested that individuals facing stressful situations are inclined to strategically utilize their voice to protect and obtain resources. The COR theory comprises both a “resource conservation” principle and a “resource accumulation” principle, which offer two conflicting predictions regarding the utilization of voice in the workplace. The “resource conservation” principle [56] posits that individuals experiencing stress may refrain from using their voice, as it entails resource expenditure. Proposing suggestions and challenging the current state carries social risks, and to ensure that their opinion is heard, employees must invest time and energy [38,57]. Hence, when employees encounter stressful situations, they may be less inclined to invest the additional time and energy required to engage in voice behavior. Conversely, the “resource acquisition” aspect of COR theory [56] suggests that individuals experiencing stress might actually increase their use of voice, viewing it as a means to acquire additional resources to mitigate stress. By voicing their concerns to supervisors or team members, employees may persuade others to provide them with extra resources to alleviate their difficulties [25].

We suggest that whether employees choose to use their voice in a stressful situation to prevent or acquire resources depends on their perception of procedural justice. Studies [58,59] have indicated that employees comprehensively assess an organizational authority’s trustworthiness by relying on their perceptions of the organization’s procedural fairness. When individuals perceive procedural justice to be low, they believe that their suggestions and ideas are not valued or actively sought by authorities in the decision-making process [52]. Employees perceiving their ideas as unlikely to be heard or believing that speaking up could result in adverse outcomes may choose not to voice them [39]. Indeed, existing studies have demonstrated that injustice in the workplace depletes individuals’ psychological resources [60,61], which leads them to prevent their resources from further depletion. On the other hand, high perceptions of procedural justice provide employees with the assurance that organizational authorities will act ethically, maintain consistency, and avoid bias. This, in turn, diminishes employees’ fear of facing detrimental consequences when suggesting ideas or opinions on work-related matters [57]. As a consequence, individuals utilize voice as an instrument to acquire additional resources that can enhance job performance and career advancement [20,62].

**Figure 1:**
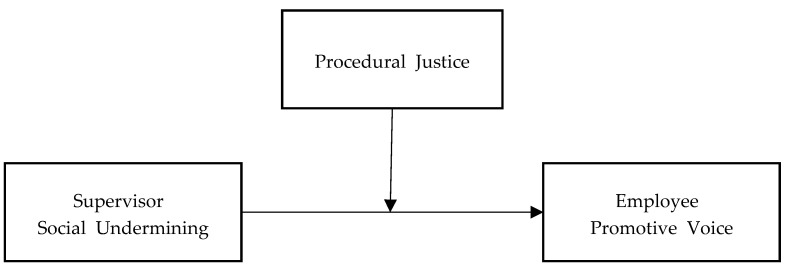
Research model.

Drawing from the aforementioned arguments, we believe that the experience of supervisor social undermining does not uniformly discourage employees from proposing constructive ideas or suggestions. Even when confronted with supervisor-driven social undermining behavior, individuals with strong perceptions of procedural justice may still choose to voice their opinions to obtain more resources because of their confidence in the fairness of organizational authority. As such, we posit that the perception of procedural justice can act as a moderating factor in the relationship between supervisors’ social undermining and employees’ promotive voices (see Figure 1). Hence, we propose the following hypotheses:

**Hypothesis** **2.** *Employee procedural justice perception moderates the negative relationship between supervisor social undermining and employee promotive voice, creating a weaker relationship when the level of procedural justice perception is high than when it is low.*

## 3. Methods

### 3.1. Sample and Procedure

We conducted a survey involving 180 full-time professional employees from manufacturing firms located in Namangan and Tashkent, Republic of Uzbekistan. The manufacturing sector companies were chosen due to their typically hierarchical organizational structure, which increases the likelihood of supervisor social undermining behavior. To collect the data, we reached out to multiple well-established companies in these cities. With management approval, we were able to proceed with data collection from employees in seven of these companies. As the use of Internet-based surveys has become widespread [63,64], we employed an online questionnaire to gather data. HR managers assisted us by distributing the questionnaire link to randomly selected employees. They also encouraged these employees to participate in the survey. Following the initial email distribution, we also asked them to send reminder emails after a one-week interval. In the cover letter of the questionnaire, we highlighted that participation in the survey was voluntary. Moreover, we reassured participants that any information they provided would be kept anonymous and confidential. Additionally, we stressed the importance of providing honest answers by clarifying that there were no correct or incorrect responses to the questions, thus ensuring the validity of the results. Participants responded to questions regarding supervisors’ social undermining, procedural justice, and employee promotive voice.

Out of the 180 responses received, we excluded 65 responses due to missing information, resulting in a total of 115 valid responses for the study, yielding a response rate of 63.8%. The majority of respondents, comprising 71.3% of the 115 participants, were men. In terms of participants’ age, the majority fell within the 25–35 and 36–45 age groups, accounting for 60.9% and 24.3%, respectively. Meanwhile, 8.7% were in the 18–25 age range, and 6.1% were between 46 and 65 years old. Regarding participants’ tenure, 19.1% had less than 3 years of experience, 20.0% had 4–5 years, 28.7% had 6–9 years, 26.1% had 10–15 years, and 6.1% had more than 15 years of work experience. Concerning the participant’s education level, the predominant level among participants was a bachelor’s degree, encompassing 73.9% of the total. Additionally, 11.3% had a high school diploma, 10.4% held a master’s degree, and 4.3% held a PhD.

### 3.2. Measures

Because the measurements were originally developed in English, we translated them into Uzbek with the assistance of professional translators. To verify the accuracy of the questionnaire translation, we conducted a back translation of the questionnaires [65].

We assessed all three variables (supervisors’ social undermining, procedural justice, and employees’ promotive voice) using items from previous empirical studies found in the relevant literature. To be specific, 13 items from Duffy et al. [17] were utilized to assess supervisor social undermining (α = 0.934). Sample items were, “Undermined your effort to be successful on the job”, “Delayed work to make you look bad or slow you down”, and “Gave you the silent treatment”. Participants assessed supervisors’ social undermining using a 5-point scale, ranging from 1 (never) to 5 (always). Procedural justice (α = 0.720) was assessed via 7 items from Colquitt [66]. Sample items were “Have you influenced the (outcome) arrived at by those procedures?” and “Have these procedures been applied consistently?” and “Have those procedures upheld ethical and moral standards?” The scale ranged from 1 (strongly disagree) to 5 (strongly agree). Employee promotive voice (α = 0.822) was measured using 5 items from Liang et al. [6]. Example items are “I proactively suggest new projects which are beneficial to the work unit”, “I make constructive suggestions to improve the unit’s operation”, and “I proactively voice out constructive suggestions that help the unit reach its goals”. Participants rated employee voice using a 5-point scale, ranging from 1 (never) to 5 (always).

Prior research [6,38] has utilized gender, age, educational level, and tenure as control variables because of their potential influence on employee voice behavior. In line with this practice and to mitigate the risk of potentially misleading associations, we also incorporated controls for gender, age, educational level, and tenure of the respondents.

## 4. Results

SPSS (version 23.0) was used for data analysis. The researcher tested the significance of the relationships between the variables and determined whether the data were appropriate for the study model [67]. The validity and reliability of each construct were evaluated using confirmatory factor analysis, and the hypotheses were tested using linear regression analysis. Prior to the analysis, common method bias was assessed using exploratory factor analysis. The analysis revealed no emergence of a single factor, and a general factor did not explain most of the variance. In fact, the largest factor accounted for 29.228% of the variance, indicating a small chance of common method bias [68].

Table 1 presents the descriptive statistics for all measured variables. A confirmatory factor analysis was used to evaluate the convergent validity, discriminant validity, and internal consistency reliability of the constructs in the measurement model. All the average variance extracted (AVE) and composite reliability (CR) values surpassed the recommended threshold of 0.50 and 0.70%, respectively, indicating acceptable convergent validity [69]. Discriminant validity was demonstrated by the average variance extracted (AVE) values, which ranged from 0.562 to 0.651, surpassing all squared correlations for each pair of constructs, which ranged from 0.062 to 0.131. The findings further indicated that composite construct reliability estimates for each construct ranged from 0.774 to 0.933, and Cronbach’s α varied from 0.720 to 0.934. As both composite reliability (CR) and Cronbach’s α values exceeding 0.70 indicate a strong level of reliability [70], internal consistency reliability is considered satisfactory in this study.

The correlation and discriminant validity tests are presented in Table 1. The correlation analysis revealed a negative correlation between supervisor social undermining and employee promotive voice (r = −0.362, *p* < 0.01), whereas procedural justice is positively correlated with employee promotive voice (r = 0.283, *p* < 0.01). These results confirm the consistency of the hypotheses and their directions.

We assessed the validity of the proposed hypotheses using a regression analysis. First, we investigated the influence of the control variables on employees’ promotive voices. The findings suggest that factors such as age, sex, tenure, and educational level do not significantly affect employees’ promotive voices. Regarding the first hypothesis proposing a negative relationship between supervisor social undermining and employee promotive voice, the analysis results reveal a significant negative association between supervisor social undermining and employee promotive voice (β = −0.357, *p* < 0.001). Hence, our findings support Hypothesis 1 (H1).

**Figure 2:**
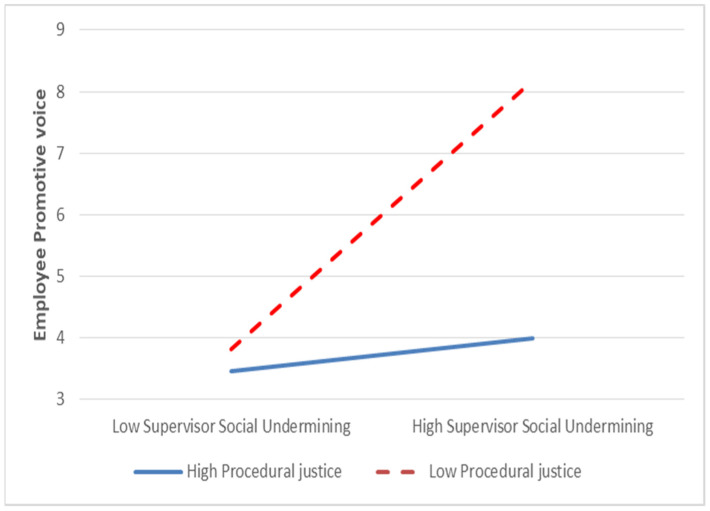
Moderating effect of procedural justice on the relationship between supervisor social undermining and employee promotive voice.

To evaluate the moderating effect of procedural justice on the relationship between supervisor social undermining and employees’ promotive voice, we performed a hierarchical regression analysis. The results of the hierarchical regression analysis (Table 2) indicate that procedural justice moderates the relationship between supervisor social undermining and employee promotive voice (β = 0.232, *p* < 0.01). However, this finding contradicts our proposed hypothesis that employee procedural justice perception moderates the negative relationship between supervisor social undermining and employee promotive voice, leading to a weaker relationship when the level of procedural justice perception is higher than when it is low. To examine the nature of the interaction, we graphed simple slopes depicting the relationship between supervisors’ social undermining and employees’ promotive voices. The results of the simple slope analysis (Figure 2) indicated that the effect of supervisors’ social undermining on employees’ promotive voice was stronger among employees with a higher perception of procedural justice. Conversely, the effect of supervisors’ social undermining on individuals’ promotive voice was weaker for those with a lower perception of procedural justice. Hence, our findings do not support Hypothesis 2 (H2).

## 5. Discussion

### 5.1. Theoretical Discussion

In this study, we investigated the association between supervisor social undermining and employee promotive voice and assessed the moderating effect of procedural justice on this relationship. This study makes various contributions to the current body of literature.

Earlier investigations [15,16] explored the link between supervisor social undermining and employee voice behavior. These studies observed that when supervisors exhibit socially undermining behavior towards employees, individuals tend to be hesitant to express their opinions and suggestions on work-related matters. Despite these scholars’ contributions to examining the impact of supervisor social undermining on general employee voice behavior or various voice forms (such as acquiescence, defense, and prosocial), there has been a notable gap in the study of promotive voice, the form of voice that distinguishes itself through its content, function, and impact on others. To address this gap, this study investigates the effect of supervisor social undermining on employees’ promotive voices. Our study revealed that when supervisors hindered their subordinates’ efforts towards success, belittled their ideas, or created a sense of incompetence, they were less inclined to actively express constructive suggestions that contributed to the unit’s goal attainment. Therefore, our study adds to the literature by presenting empirical evidence of the adverse impact of supervisor social undermining on a crucial innovative form of employee voice, specifically promotive voice.

Second, prior research suggested that when employees encounter workplace mistreatment such as social undermining behavior, they tend to avoid speaking up and sharing information [15,71,72]. Nevertheless, multiple studies have highlighted that workplace mistreatment affects employees differently [9,11], and scholars have proposed that organizational factors, particularly procedural justice perception, play a significant role in determining whether employees decide to express their opinions or remain silent [22]. Thus, employees’ perception of procedural justice may influence the connection between supervisors’ social undermining and employees’ promotive voices. However, no studies have explored whether procedural justice mitigates the adverse impact of supervisors’ social undermining on employees’ promotional voices. To fill this research gap, our study investigates the moderating role of procedural justice in the relationship between supervisors’ social undermining and employees’ promotive voices. Specifically, we proposed that the link between supervisor social undermining and employee promotive voice will be less strong when employees perceive a high level of procedural justice compared to when their perception of procedural justice is low. Contrary to our expectations, the findings suggest that the perception of procedural justice strengthens the association between supervisors’ social undermining and employees’ promotive voice. These contradictory findings could be attributed to several factors. For instance, Duffy et al. [17] demonstrated in their study that negative events exert a stronger influence on employee outcomes than positive ones. Their research revealed that when employees perceive social undermining and support from their supervisors, supervisor support strengthens the negative effects of supervisor social undermining on employee job outcomes. Hence, even though the sources differ in our study, it is conceivable that when employees perceive a high level of procedural justice (positive events), they become more sensitive to discrepancies or inconsistencies in their supervisors’ behavior, such as instances of social undermining (negative events). Consequently, instances of social undermining may become more salient, resulting in a more pronounced negative impact on the promotive voice.

Moreover, according to Huang and Huang [73], individuals tend to exhibit more discretionary behaviors when they perceive fair interpersonal treatment from authority figures and perceive organizational procedures as fair. In contrast, when employees perceive low procedural justice, the adverse association between interactional justice and employee tendency to withhold suggestions and concerns will be weaker because the two justice dimensions could demonstrate conflicting information for the employee’s justice judgment. Hence, it is reasonable to infer that when employees perceive negative interactional justice, such as social undermining, their high perception of procedural justice amplifies the adverse impact of supervisor social undermining on promotive voice behavior. Consequently, our study makes a valuable contribution to the literature by offering empirical evidence on the moderating role of procedural justice in supervisor social undermining–employee promotive voice relationships, with the finding that employee perception of procedural justice may strengthen the link between supervisor social undermining and employees’ promotive voice.

### 5.2. Managerial Implications

Drawing on our findings, we propose several practical implications for organizational managers. For instance, the findings indicate that when supervisors engage in social undermining behavior, employees tend to hesitate to offer constructive opinions and ideas aimed at enhancing the performance of their work unit or organization. These findings broaden our understanding of organizational management by highlighting supervisor behaviors that hinder employees’ promotive voices, a crucial element in fostering innovation and successful adjustment to competitive business environments. With this insight, organizational managers can recognize the significance of managing and mitigating supervisors’ social undermining behavior. We suggest that organizations can mitigate supervisory social undermining behaviors through personnel management practices. These practices should provide training and development initiatives to foster ethical perspectives on managerial responsibilities among supervisors [74]. Clear expectations and policies regarding social undermining should be communicated effectively [75]. Organizational leaders should convey the consequences of such detrimental workplace behaviors and establish strict policies against mistreatment [17]. Moreover, managers may foster a promotive voice among employees by ensuring that they perceive themselves as integral contributors to the organization’s development. This can be achieved through superiors actively listening to employees, encouraging the suggestion of innovative ideas to enhance the work environment, and involving individuals in decision-making processes [11].

Furthermore, our findings revealed that when employees perceive high levels of procedural justice, the adverse impact of supervisors’ social undermining on their promotive voices intensifies. These findings emphasize the crucial importance of supervisors’ social undermining behavior, particularly in environments where procedures are perceived to be conducted fairly. Despite organizations implementing decision-making procedures fairly, consistently, without bias, and with openness to employee input, supervisors’ social undermining behavior counteracts the positive effects of these efforts, which might otherwise encourage employees to engage in more voice behavior. Therefore, organizations should address various instances of social undermining that may arise in the workplace to promote the transparent and fair treatment of employees. Management of organizations should identify perpetrators, administer anonymous surveys to collect information to promptly prevent the detrimental effects of undermining, and establish guidelines regarding acceptable behavioral norms [16]. These behavioral expectations can be integrated into supervisory performance evaluations to offer regular feedback to supervisors regarding their professional development [15]. Additionally, organizational management should assess their organizational culture, as it significantly impacts the prevalence of employee social undermining behavior [16]. By understanding these dynamics, organizations can work to create a positive culture that discourages social undermining and fosters supportive, collaborative behaviors, thereby encouraging employees to engage more in promotive voice behaviors.

### 5.3. Limitations and Future Research Directions

The present study has some limitations, highlighting important avenues for future research. First, while our study focused solely on supervisor social undermining as a driver of employee promotive voice, it’s important to acknowledge that other factors, such as coworker social undermining, can also significantly influence employees’ engagement in voice behavior. Therefore, future investigations should include coworker social undermining and compare the impact of supervisor and coworker social undermining on employees’ promotive voices. Second, this study concentrated solely on employee procedural justice perception as a moderator between supervisor social undermining and employee promotive voice. We recommend future studies to examine the moderating role of organizational culture, as it is an important factor influencing employee voice behavior. Future studies may expand their scope to include organizational and individual factors as moderators. For example, previous research has highlighted the impact of an individual’s emotion regulation ability. Therefore, we recommend that future studies consider emotion regulation strategies, such as problem-focused or emotion-focused approaches, as moderating variables when exploring the relationship between supervisor social undermining and employee promotive voice. Third, previous research has shown that a supervisor’s social undermining behavior can lead to subordinates becoming cynical, and cynical employees are less likely to engage in voice behavior. Thus, we suggest that future research investigate whether employee cynicism can explain the link between supervisor social undermining and employee promotive voice. Moreover, examining whether organizational culture or perceptions of procedural justice moderate the mediating effect of employee cynicism on the relationship between supervisor social undermining and employee voice can broaden the understanding of these dynamics. Fourth, our study was conducted only in the Republic of Uzbekistan, which potentially limits the generalizability of the results. We suggest replicating similar studies in multiple countries to enhance the broader applicability of our findings. Additionally, to enhance the generalizability of the findings, future research should focus on increasing the sample size. By broadening the dataset, researchers can encompass a broader spectrum of viewpoints and encounters, thereby fostering a holistic comprehension of the phenomena being studied. Furthermore, a larger sample size facilitates statistical analyses with increased power and reliability, thereby facilitating more precise conclusions. Hence, forthcoming studies are urged to enroll larger samples in their investigations.

## 6. Conclusions

Although earlier research has studied the impact of social undermining on employees speaking up, the relationship between supervisor social undermining and employee promotive voice—an innovative type of voice behavior—remained uncertain. Our study demonstrated that supervisor social undermining reduces employees’ willingness to engage in a promotive voice. Moreover, we discovered that employee perception of procedural justice moderates this negative relationship. Specifically, when employees perceive high procedural justice, the negative impact of social undermining on promotive voice is stronger, while it is weaker when perceptions of procedural justice are low.

## Figures and Tables

**Table 1:** Means, standard deviations, and bivariate correlations for variables.

Variable	Mean	SD	1	2	3	4	5	6	7
1. Gender	0.713	0.454	1						
2. Age	2.287	0.734	0.091	1					
3. Education level	2.078	0.623	−0.044	0.429 **	1				
4. Tenure	3.252	1.626	0.039	0.497 **	0.067	1			
5. Supervisor Social Undermining	1.667	0.799	−0.187 *	0.049	0.109	0.055	1		
6. Procedural Justice	2.628	0.851	0.028	0.095	0.026	0.176	−0.250 **	1	
7. Employee Promotive Voice	3.706	0.854	0.151	0.060	0.063	0.000	−0.362 **	0.283 **	1
AVE							0.610	0.562	0.651
CR							0.933	0.774	0.903

* *p* < 0.05; ** *p* < 0.01.

**Table 2:** Results for hierarchical moderated regression analysis for procedural justice.

	Model 1	Model 2	Model 3	Model 4
Gender	0.152	0.086	0.092	0.063
Age	0.036	0.034	0.034	0.009
Education level	0.056	0.092	0.083	0.081
Tenure	−0.028	−0.007	−0.047	−0.004
Supervisor social undermining		−0.357 ***	−0.301 ***	−0.218 *
Procedural justice			0.208 *	0.244 **
interaction: Super. Soc. Under. X Proced. Justice				0.232 **
R^2^	0.029	0.150	0.189	0.233
Adjusted R^2^	−0.007	0.111	0.144	0.183
Change in R^2^	0.029	0.121	0.039	0.044
F	0.813	3.853	4.199	4.637

Dependent variable: Employee Promotive Voice. * *p* < 0.05; ** *p* < 0.01; *** *p* < 0.001.

## Data Availability

Data are contained within the article.

## References

[1] Shin D., Woodwark M.J., Konrad A.M., Jung Y. (2022). Innovation strategy, voice practices, employee voice participation, and organizational innovation. J. Bus. Res..

[2] Lee J., Choi D., Cheong M. (2023). Leader Boundary-Spanning Behavior and Employee Voice Behavior: The Job Demands–Resources Perspective. Behav. Sci..

[3] Huang X., Van de Vliert E., Van der Vegt G. (2005). Breaking the silence culture: Stimulation of participation and employee opinion withholding cross-nationally. Manag. Organ. Rev..

[4] Van Dyne L., Ang S., Botero I.C. (2003). Conceptualizing employee silence and employee voice as multidimensional constructs. J. Manag. Stud..

[5] Lin S.H.J., Johnson R.E. (2015). A suggestion to improve a day keeps your depletion away: Examining promotive and prohibitive voice behaviors within a regulatory focus and ego depletion framework. J. Appl. Psychol..

[6] Liang J., Farh C.I.C., Farh J.-L. (2012). Psychological antecedents of promotive and prohibitive voice: A two wave examination. Acad. Manag. J..

[7] Fan P., Liu Y., Liu H., Hou M. (2022). The multilevel influence of supervisor helping behavior on employee voice behavior: A moderated mediation model. Front. Psychol..

[8] Detert J.R., Treviño L.K. (2010). Speaking up to higher-ups: How supervisors and skip-level leaders influence employee voice. Organ. Sci..

[9] Dedahanov A.T., Fayzullaev A.K.U., Abdurazzakov O.S. (2022). Supervisor incivility and employee voice: The roles of cognitive reappraisal and psychological distress. Leadersh. Organ. Dev. J..

[10] Rani H., Shah S.M.M., Umrani W.A., Syed J., Afshan G. (2021). Employee state paranoia: Linking abusive supervision with employee voice behavior. Leadersh. Organ. Dev. J..

[11] Sun W., Dedahanov A.T., Fayzullaev A.K.U., Abdurazzakov O.S. (2022). Abusive supervision and employee voice: The roles of positive reappraisal and employee cynicism. Front. Psychol..

[12] Liu C.E., Chen W.X.Y., Huang J. (2018). Supervisor incivility and employee silence: Does Chinese traditionality matter. Int. J. Bus. Soc. Sci..

[13] Li C.-F., Tian Y.-Z. (2016). Influence of workplace ostracism on employee voice behavior. Am. J. Math. Manag. Sci..

[14] Liu S., Sun M., Guan Y.-W., Liu C.-Y., Ren B., Yang Q. (2023). How leadership ostracism influences public servants’ promotive voice: Public service motivation as a moderator. Soc. Behav. Personal..

[15] Frazier M.L., Bowler W.M. (2015). Voice climate, supervisor undermining, and work outcomes: A group-level examination. J. Manag..

[16] Jung H.S., Yoon H.H. (2019). The effects of social undermining on employee voice and silence and on organizational deviant behaviors in the hotel industry. J. Serv. Theory Pract..

[17] Duffy M.K., Ganster D.C., Pagon M. (2002). Social undermining in the workplace. Acad. Manag. J..

[18] Qin X., DiRenzo M.S., Xu M., Duan Y. (2014). When do emotionally exhausted employees speak up? Exploring the potential curvilinear relationship between emotional exhaustion and voice. J. Organ. Behav..

[19] Mo S., Shi J. (2016). The voice link: A moderated mediation model of how ethical leadership affects individual task performance. J. Bus. Ethics.

[20] Fuller J.B., Barnett T., Hester K., Relyea C., Frey L. (2007). An exploratory examination of voice behavior from an impression management perspective. J. Manag. Issues.

[21] Hsiung H.H. (2012). Authentic leadership and employee voice behavior: A multi-level psychological process. J. Bus. Ethics.

[22] Tangirala S., Ramanujam R. (2008). Employee silence on critical work issues: The cross level effects of procedural justice climate. Pers. Psychol..

[23] Colquitt J.A. (2004). Does the justice of the one interact with the justice of the many? Reactions to procedural justice in teams. J. Appl. Psychol..

[24] Hobfoll S.E. (2002). Social and psychological resources and adaptation. Rev. Gen. Psychol..

[25] Ng T.W.H., Feldman D.C. (2012). Employee voice behavior: A meta-analytic test of the conservation of resources framework. J. Organ. Behav..

[26] Hershcovis M.S. (2011). “Incivility, social undermining, bullying...oh my!”: A call to reconcile constructs within workplace aggression research. J. Occup. Health Psychol..

[27] Greenbaum R.L., Mawritz M.B., Eissa G. (2012). Bottom-line mentality as an antecedent of social undermining and the moderating roles of core self-evaluations and conscientiousness. J. Appl. Psychol..

[28] Neuman J., Baron R., Giacalone R., Greenberg J. (1997). Aggression in the workplace. Antisocial Behavior in Organizations.

[29] Song Y., Zhao Z. (2022). Social undermining and interpersonal rumination among employees: The mediating role of being the subject of envy and the moderating role of social support. Int. J. Environ. Res. Public Health.

[30] Duffy M.K., Ganster D.C., Shaw J.D., Johnson J.L., Pagon M. (2006). The social context of undermining behavior at work. Organ. Behav. Hum. Decis. Process..

[31] Duffy M.K., Scott K.L., Shaw J.D., Tepper B.J., Aquino K. (2012). A Social Context Model of Envy and Social Undermining. Acad. Manag. J..

[32] Van Dyne L., LePine J.A. (1998). Helping and voice extra-role behaviors: Evidence of construct and predictive validity. Acad. Manag. J..

[33] Van Dyne L., Cummings L.L., McLean Parks J. (1995). Extrarole behaviors: In pursuit of construct and definitional clarity (a bridge over muddied waters). Res. Organ. Behav..

[34] Morrison E.W. (2011). Employee voice behavior: Integration and directions for future research. Acad. Manag. Ann..

[35] Ward A.-K., Ravlin E.C., Klaas B.S., Ployhart R.E., Buchan N.R. (2016). When do high-context communicators speak up? Exploring contextual communication orientation and employee voice. J. Appl. Psychol..

[36] Morrison E.W. (2023). Employee voice and silence: Taking stock a decade later. Annu. Rev. Organ. Psychol. Organ. Behav..

[37] Dedahanov A.T., Rhee C., Yoon J. (2017). Organizational structure and innovation performance: Is employee innovative behavior a missing link?. Career Dev. Int..

[38] Detert J.R., Burris E.R. (2007). Leadership behavior and employee voice: Is the door really open?. Acad. Manag. J..

[39] Burris E.R., Detert J.R., Chiaburu D.S. (2008). Quitting before leaving: The mediating effects of psychological attachment and detachment on voice. J. Appl. Psychol..

[40] Blau P.M. (2017). Exchange and Power in Social Life.

[41] Eisenberger R., Cotterell N., Marvel J. (1987). Reciprocation ideology. J. Personal. Soc. Psychol..

[42] Ilies R., Nahrgang J.D., Morgeson F.P. (2007). Leader-member exchange and citizenship behaviors: A meta-analysis. J. Appl. Psychol..

[43] Podsakoff P.M., MacKenzie S.B., Paine J.B., Bachrach D.G. (2000). Organizational citizenship behaviors: A critical review of the theoretical and empirical literature and suggestions for future research. J. Manag..

[44] Lee K., Kim E., Bhave D.P., Duffy M.K. (2016). Why victims of undermining at work become perpetrators of undermining: An integrative model. J. Appl. Psychol..

[45] Yoo J., Frankwick G.L. (2013). Exploring the impact of social undermining on salesperson deviance: An integrated model. J. Pers. Sell. Sales Manag..

[46] Tepper B.J., Carr J.C., Breaux D.M., Geider S., Hu C., Wie H. (2009). Abusive supervision, intentions to quit, and employees’ workplace deviance: A power/dependence analysis. Organ. Behav. Hum. Decis. Process..

[47] Zellars K.L., Tepper B.J., Duffy M.K. (2002). Abusive supervision and subordinates’ organizational citizenship behavior. J. Appl. Psychol..

[48] Leventhal G.S., Gergen K., Greenberg M., Willis R. (1980). What should be done with equity theory? New approaches to the study of fairness in social relationships. Social Exchange: Advances in Theory and Research.

[49] Dietz J., Robinson S.L., Folger R., Baron R.A., Schulz M. (2003). The impact of community violence and an organization’s procedural justice climate on workplace aggression. Acad. Manag. J..

[50] Liao H., Rupp D. (2005). The impact of justice climate and justice orientation on work outcomes: A cross-level multifoci framework. J. Appl. Psychol..

[51] Pinder C.C., Harlos K.P. (2001). Employee silence: Quiescence and acquiescence as responses to perceived injustice. Res. Pers. Hum. Resour. Manag..

[52] Tyler T.R., Lind E.A. (1992). A relational model of authority in groups. Adv. Exp. Soc. Psychol..

[53] Chou S.Y., Barron K. (2016). Employee voice behavior revisited: Its forms and antecedents. Manag. Res. Rev..

[54] Zhang L., Lou M., Guan H. (2022). How and when perceived leader narcissism impacts employee voice behavior: A social exchange perspective. J. Manag. Organ..

[55] Ahmad B., Shafique I., Kalyar M.N. (2022). A moderated mediation model of the association between coworker social undermining and knowledge hiding. VINE J. Inform. Knowl. Manag. Syst..

[56] Hobfoll S.E. (1989). Conservation of resources: A new attempt at conceptualizing stress. Am. Psychol..

[57] Luria G., Gal I., Yagil D. (2009). Employees’ willingness to report service complaints. J. Serv. Res..

[58] Bos K.v.D., Vermunt R., Wilke H.A.M. (1997). Procedural and distributive justice: What is fair depends more on what comes first than on what comes next. J. Personal. Soc. Psychol..

[59] Van den Bos K., Lind E.A., Wilke H.A., Cropanzano R. (2001). The psychology of procedural and distributive justice viewed from the perspective of fairness heuristic theory. Justice in the Workplace.

[60] Cole M.S., Bernerth J.B., Walter F., Holt D.T. (2010). Organizational justice and individuals’ withdrawal: Unlocking the influence of emotional exhaustion. J. Manag. Stud..

[61] Wang H.-J., Lu C.-Q., Siu O.-L. (2015). Job insecurity and job performance: The moderating role of organizational justice and the mediating role of work engagement. J. Appl. Psychol..

[62] Seibert S.E., Kraimer M.L., Crant J.M. (2001). What do proactive people do? A longitudinal model linking proactive personality and career success. Pers. Psychol..

[63] Jing E.L., Gellatly I.R., Feeney J.R., Inness M. (2022). Social Undermining and Three Forms of Organizational Commitment. J. Personal. Psychol..

[64] Chen J., May D.R., Schwoerer C.E., Deeg M. (2023). “Called” to speak out: Employee career calling and voice behavior. J. Career Dev..

[65] Brislin R. (1993). Understanding Culture’s Influence on Behavior.

[66] Colquitt J.A. (2001). On the dimensionality of organizational justice: A construct validation of a measure. J. Appl. Psychol..

[67] Byrne B.M. (2010). Structural Equation Modeling with AMOS.

[68] Podsakoff P.M., MacKenzie S.B., Lee J.-Y., Podsakoff N.P. (2003). Common method biases in behavioral research: A critical review of the literature and recommended remedies. J. Appl. Psychol..

[69] Fornell C., Larcker D.F. (1981). Evaluating structural equation models with unobservable variables and measurement errors. J. Mark. Res..

[70] Nunnally J.C. (1978). Psychometric Theory.

[71] Afshan G., Kashif M., Sattayawaksakul D., Cheewaprakobkit P., Wijenayake S. (2022). Abusive supervision, supervisor undermining, and turnover intentions: Mediation of quiescent silence and desire to seek revenge among Thai banking frontliners. Manag. Res. Rev..

[72] Khan M.A., Malik O.F., Shahzad A. (2022). Social undermining and employee creativity: The mediating role of interpersonal distrust and knowledge hiding. Behav. Sci..

[73] Huang L., Huang W. (2016). Interactional justice and employee silence: The roles of procedural justice and affect. Soc. Behav. Personal..

[74] Bouichou S.I., Wang L., Feroz H.M.B. (2022). How corporate social responsibility perceptions affect employees’ positive behavior in the hospitality industry: Moderating role of responsible leadership. Int. Rev. Public Nonproft Mark..

[75] Eissa G., Wyland R., Gupta R. (2020). Supervisor to coworker social undermining: The moderating roles of bottom-line mentality and self-efficacy. J. Manag. Organ..

